# Illuminating innovations: a conversation with René-Jean Essiambre on the frontiers of optical communication

**DOI:** 10.1038/s41377-025-02165-6

**Published:** 2026-03-02

**Authors:** Yating Wan, Chunxiu Zang

**Affiliations:** 1 Integrated Photonics lab, King Abdullah University of Science and Technology, Thuwal, 23955-6900 Kingdom of Saudi Arabia; 2 Changchun Institute of Optics, Fine Mechanics and Physics, Chinese Academy of Sciences, Changchun, 130033 China

**Keywords:** Optoelectronic devices and components, Fibre optics and optical communications

## Abstract

In the rapidly evolving digital era, optical communication plays a vital role, serving as the foundational technology behind our connected world, from high-speed internet to global telecommunication networks. At the center of this field, Dr. René-Jean Essiambre ‘s contributions have been significant. He is known for developing the nonlinear Shannon limit theory, which has helped the industry better understand the impact of nonlinearity on optical fiber capacity. This work is important for designing today’s high-speed optical networks and lays the groundwork for future innovations.

In this exclusive interview, Dr. Essiambre shares insights into his journey, the challenges and opportunities in the field, his views on the future of optical communication and offers advice to young researchers. Join us as we delve into the thoughts of one of the leading figures in optical communication and gain a glimpse into the future in this dynamic and rapidly evolving field. For more details on Dr. Essiambre’s experiences and advice, the full interview is available in the Supplementary Information.

**Figure:**
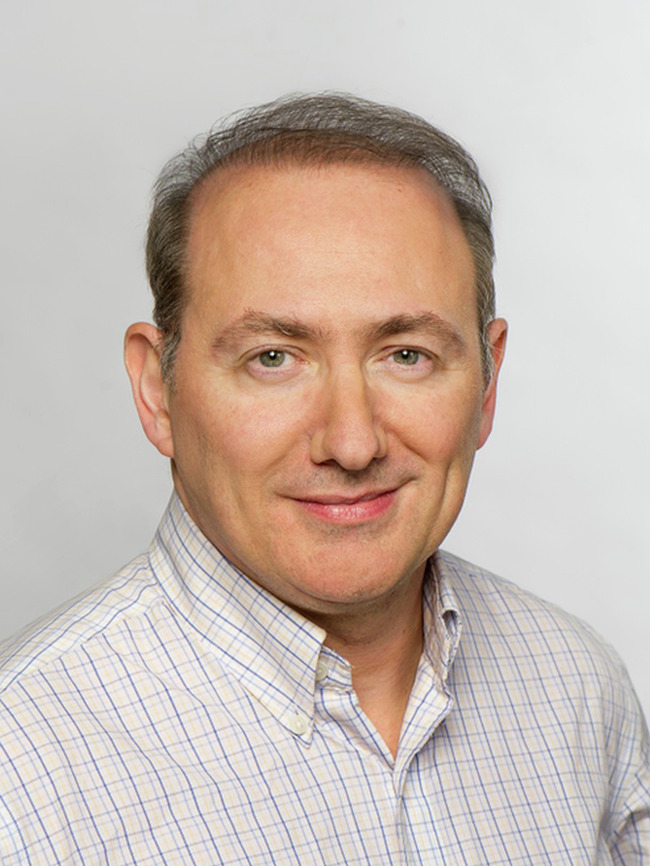

**Short Bio:** Dr. René-Jean Essiambre is a Distinguished Member of Technical Staff (DMTS) and a Bell Labs Fellow at Nokia Bell Labs (Bell Labs was the Research Laboratory of AT&T, which then became part of Lucent Technologies, followed by Alcatel-Lucent and is now part of Nokia since 2016). He received the Ph.D. degree in physics (optics) from Laval University in 1994. During his doctoral studies, he spent a year studying solid-state physics at McGill University. He pursued post-doctoral studies in optical communication at the Institute of Optics, University of Rochester. Dr. Essiambre is a Fellow of the Institute of Electrical and Electronics Engineers (IEEE), Optica (formerly OSA), the Institute of Advanced Studies at Technical University of Munich (IAS-TUM), where he is also an Ambassador of the University. Dr. Essiambre was a member of the Board of Governors and President of the IEEE Photonics Society. He is a recipient of the 2005 Engineering Excellence Award from OSA (now Optica). He works on fiber lasers, nonlinear optics in fibers, coherent detection, advanced modulation formats, space-division multiplexing, information theory applied to optical fibers, and quantum communication. He participated in the design and some of his inventions have been implemented in commercial fiber-optic communication systems.

**Q1: You have a long-standing expertise in optics and your work in optical communications has significantly influenced the industry. Could you briefly describe your current research focus and what initially attracted you to this field?**

**A1:** Sure, my current research is quite different from what I’ve done in the last 20 years. I’m now focused on quantum technologies for optical communication, which is still mostly in the early experimental phase. My most immediate goal is to more closely approach the Gordon-Holevo limit, a theoretical bound on the maximum rate of transmission of classical information through a quantum channel. This involves incorporating the quantum properties of light to enhance optical system capacity. While the theoretical derivation of this capacity limit has been performed, figuring out a way to achieve it remains a challenge. This is one of the few capacity bounds in optical communication that has yet to be fully explored, and I’m working to push the limits using quantum technologies.

Previously, I spent many years working to increase the capacity of classical optical systems, especially through managing nonlinearities in fibers and applying information theory to approach the Shannon limit using classical light and coherent detection. In around 2006, we came up with a more precise estimation of the fiber capacity limits that we published in 2010. Since then, we have transitioned from single mode to exploring multimode and multicore fibers. With substantial progress having been made in this area, reaching a point close to maturity in research, the next step was clear: delving into the quantum aspects of light to discover new possibilities in the field.

**Figure:**
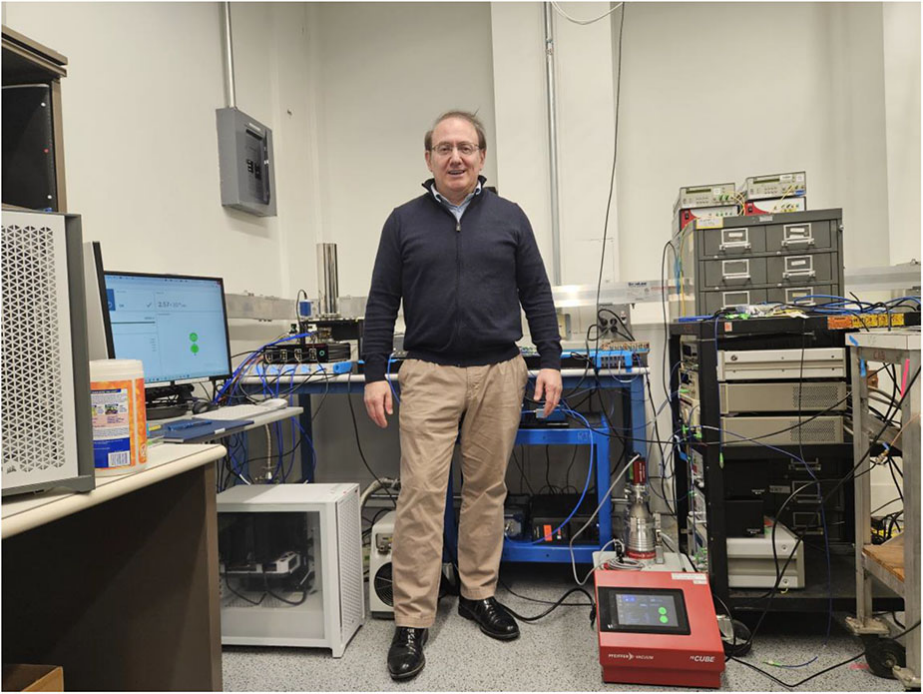
Dr. René-Jean Essiambre in front of the Single Quantum photon detectors

**Q2: Your initial academic focus was on astrophysics, then you transitioned to optical communication after completing your master’s studies. What inspired this shift and how has your background in astrophysics influenced your approach in optics?**

**A2:** My academic background is in physics, with a particular interest in astrophysics. During my master’s, I worked on understanding the distribution and inherent structural patterns of galaxies and how they were influenced by gravity. This involved characterizing the fractal nature of the distribution of galaxies that resulted from coupled propagation equations describing gravitational attraction between galaxies. This experience with nonlinear dynamics sparked my interest in similar complex theories within other fields of physics.

Following my master’s, I realized the limited job prospects in astrophysics at that time and decided to explore other areas within physics that had better career opportunities. This led me to solid-state physics at McGill University. Although it was a valuable experience, I found that my true passion is elsewhere. With the strong optics research community in Québec City, I shifted my focus to nonlinear fibers in optical communication. This field, while distinct, shared conceptual parallels with astrophysics particularly in dealing with nonlinearities. The shift allowed me to stay engaged in my scientific interests and opened up new career possibilities. My work in optics has been very rewarding, confirming that it was the right decision for me.

**Q3: Your research endeavors span from astrophysics, nonlinear optical communications, and now quantum communications. Among your numerous projects, which achievements stand out most to you?**

**A3:** It’s hard to single out one achievement, but a defining moment was in the late 1990s at Bell Labs. At the time, the common wisdom was that the signal in high-speed fiber transmission would suffer greatly from the combined effect of fiber chromatic dispersion and nonlinearity, making solitons or alternative methods necessary. Despite this, I decided to explore what could be achieved with high-speed transmission through simulation and experimentation. This led to the discovery of a new nonlinear transmission regime that allowed for high power levels and reduced the deleterious effects of dispersion by arranging the fiber in a particular order.

This breakthrough led to a new regime known as “Pseudo-linear transmission”, which introduced a different approach to handling nonlinearities in the next generation of Wavelength Division Multiplexing (WDM) systems. This discovery helped in designing commercial systems that could transmit at speeds of 40 Gb per second per WDM channel, which was significant at the time.

I’m also proud of my work on the nonlinear Shannon limit in fibers, which synthesized a decade of knowledge and collaboration, especially with information theorists Gerard Foschini and Gerhard Kramer. Their unique perspectives were incredibly rewarding, and on a personal level, their friendship is something I deeply value.

Another notable work is digital back-propagation, where I was among the first to publish findings on reversing nonlinear effects digitally at the receiver or the transmitter. Furthermore, my experience with the design of optical fibers and research on nonlinear propagation enabled me to foresee that coupled-core fiber technology would be one of the best fibers supporting multiple modes of propagation. Looking ahead, I’m excited about my ventures into harnessing the quantum nature of light to improve communications. Although it’s a new research area for me, I’m eager to explore and make meaningful contributions to this evolving field.

**Q4: What potential impacts do you foresee in quantum communication?**

**A4:** Quantum communication has different meanings to different people. There is currently a strong focus on quantum networks, broadly defined as optical networks that allow transport and storage of specially prepared quantum states of light. A challenge in these networks is that quantum states gradually lose their quantum properties when they encounter increasing loss, significantly altering their information content. Up to now, my efforts have focused on maximizing classical information transport using quantum means and achieving highly sensitive detection in channels with high attenuation. For example, we are exploring how to preserve high information content even under severe propagation circumstances, which could be critical for applications like deep-space exploration. Recently, we achieved what, to our knowledge, is the lowest energy-per-bit detection in the infrared and optical spectra for these types of high-attenuation channels. This achievement could enable communication over great distances in deep space or through any other highly lossy channel. We are currently investigating the generation of special quantum states to further improve these systems.

Transitioning from traditional optical communication methods to introducing quantum technologies is challenging and involves significant risks due to the complexity introduced and the high degree of uncertainty about outcomes. However, this challenge is exciting. At Nokia Bell Labs where I work, there is strong support for fundamental research that can impact communication and therefore has potential for commercial applications, directly or through novel technology developed in the process. They provide the necessary resources and encourage exploration of new frontiers, which is vital for pursuing these innovative approaches.

**Q5: In 2010, you shifted your research focus towards fibers supporting multiple spatial modes, such as multicore and multimode fibers. Could you talk more about this transition?**

**A5:** Sure, around 2006, we initiated a specific study on the capacity limit of single-mode fiber. By 2010, we had a solid estimation of these limits. Nokia Bell Labs is a multidisciplinary environment, blending expertise from wireless, optical, and network systems. During this period, our colleagues in wireless communication were implementing MIMO (Multiple Input, Multiple Output) communication, which utilizes multiple antennas to increase capacity while keeping the same total transmit power level. Inspired by this and based on advice from wireless and information theory colleagues, we considered a similar approach for optical fibers.

Although optical cables with many individual fibers had already been developed, the concept of integrating these fiber bundles into a single multicore fiber presented new possibilities. There are essentially two categories of multicore fibers: uncoupled and coupled cores. In uncoupled cores, each core is isolated enough to prevent signal leakage (also called crosstalk) to adjacent cores. In coupled-core fibers, however, the cores are close enough that injecting power into one eventually leads to the power spreading over all cores.

My Ph.D. thesis focused on two-core coupled-core fibers, exploring their advantages for nonlinear transmission. My involvement with our optical fiber division during my first two years at Bell Labs made me understand that coupled-core fibers should be easier to fabricate and to operate than uncoupled-core fibers. However, a significant challenge with coupled-core fibers is the need to process all cores modes jointly within a single large electronic processor. Despite this challenge, we decided to proceed and collaborate with fiber manufacturing companies to develop these fibers, specifically for nonlinear transmission applications and high core density.

This shift also led Bell Labs to investigate multimode fibers, which could potentially achieve even higher capacities due to their high mode density. My work in this area spanned about a decade from 2010 to 2019, and research in this field continues to evolve.

**Q6: Your work blends theoretical and experimental methods. Could you share some memorable challenges you’ve encountered and how you addressed them?**

**A6:** I like both theory and experiments, and my preference often depends on the topic. My theoretical grounding in nonlinearities was significantly enhanced by my studies with Govind Agrawal, a leading figure in the field, who served as one of my mentors for nearly three years and even today.

At Bell Labs, there was a significant demand for theoretical expertise to address system limitations caused by fiber nonlinearity —a complex challenge crucial for new system developments. After a key theorist in our department retired, I filled this gap for more than a decade, focusing on the theory to meet the business demand. One practical challenge was achieving the first 2.5 Gb per second per wavelength transmission using dispersion mapping. This technique uses fibers with positive dispersion to offset the negative dispersion of transmission fibers. This design, known as a dispersion map, was also scalable to 10 Gb per second systems, which we later commercialized.

As the technology evolved towards multicore fibers, multimode fibers, and few-mode fibers to enhance capacity efficiency, I shifted to experimental work to explore aspects such as mode index differences and phase variations in few-mode and multimode fibers. These elements required experimental exploration because of the limitations on how practical fibers behave.

My approach is to focus on the best technique to learn critical information: I turn to experimentation when exploring unknowns to gain physical insights. Once the physical behavior is well defined, I generally shift to theoretical methods, which offer a structured way to optimize or find new solutions difficult to explore experimentally. For instance, the equations for nonlinear propagation in single mode fiber are very accurate, making theoretical analysis the most logical approach once the model is established. This philosophy of alternating between theory and experimentation allows me to most effectively tackle the challenges that arise in the evolution of optical communication.

**Q7: Could you talk about the interplay between industry and academia? How can these sectors contribute to each other?**

**A7:** The relationship between industry and academia is both complex and mutually beneficial. In industry, the primary focus is on producing the best possible devices and systems with optimal performance for market success. For example, at Nokia Bell Labs, we strive to support Nokia’s business units while also engaging in foundational research that addresses potential challenges. In academia, there is also a blend of industry collaboration and fundamental research, influenced largely by the priorities of funding agencies as well. Collaborations between academia and industry are crucial because they bring together diverse expertise and perspectives. A practical way to foster this collaboration is through student involvement in industrial projects, which most often benefits both the students and their academic mentors.

It’s important to recognize that personal relationships also play a crucial role in the success of these partnerships. When researchers in industry and academia share work methods and ethical values, and are familiar with each other, it greatly motivates collaborative efforts. However, challenges do occur, such as when industry may overlook fundamental insights that could lead to innovation, or when academics may not fully grasp practical constraints of implementation or existing solutions already present in the industry.

Ultimately, successful collaboration hinges not only on the topic but also on strong personal connections and a mutual understanding. This dynamic interaction between academia and industry contributes significantly to advancing knowledge and technology.

**Q8: In 1997, you joined Bell Labs. What motivated you to make this important transition from academia to industry? Why did you choose to join Bell Labs?**

**A8:** I read many of Bell Labs’ papers during my graduate studies, and my interest actually began even earlier, back in Canada, when I was reading a physics book as a child. I saw a picture of the horn antenna which was used to discover cosmic background radiation by Bell Labs’ two researchers, Robert Wilson and Arno Penzias. I knew I wanted to be there from that moment. When I began my graduate studies, the papers in optical communication I was reading were mostly from Bell Labs, which reinforced my desire to work there.

After Govind Agrawal accepted me as a postdoctoral researcher at the Institute of Optics, University of Rochester, I expressed my interest in working at Bell Labs. Although I initially didn’t get a position in one of my areas of expertise during my first interview at Murray Hill, I was later redirected to Holmdel’s lab, which was even more aligned with my field and interests. Interestingly, they offered me a position in the same small building where I saw the antenna picture in my physics book when I was 12 years-old, the evidence of the Big Bang was discovered there. It’s interesting how things unfolded because I had always admired the work of Bell Labs and aspired to join the organization. There was also an element of luck involved, as the timing coincided with organizational changes at Lucent Technologies, where Bell Labs was located at the time, which opened up new opportunities.

**Figure:**
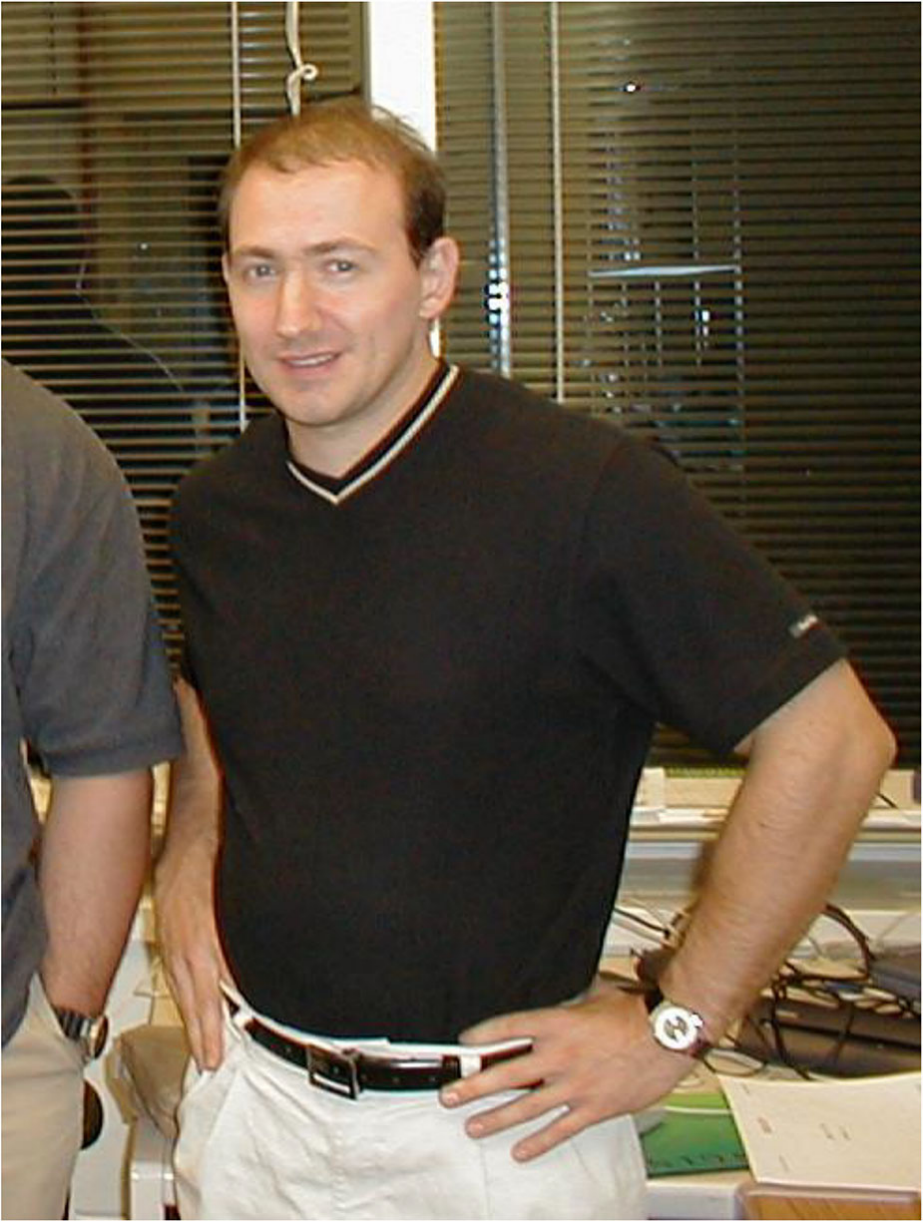
Dr. René-Jean Essiambre in his office at the Crawford Hill Laboratory in Holmdel, New Jersey

**Q9: Could you describe a typical day for you at Nokia Bell Labs? And how do you balance your project demands and creative research?**

**A9:** It’s a very good question. Throughout one’s career, you may be involved in specific projects, either internal to Nokia’s business unit or external funded by outside companies or agencies. When you’re on such a project, your focus is quite intense and specific. Most of the time, however, you work on making progress in your research area. The key is to stay self-motivated. For me, I get inspiration from discussing with colleagues and reading great articles.

Currently, on a typical day, I spend time thinking about new ideas on using quantum technologies, exploring them numerically, theoretically, or experimentally. I set up experiments, sometimes with students, to prepare materials for them and myself. During the summer, I’m in the lab every day, working with students and guiding them on what they need to know to achieve the goals we set. Personally, I like setting goals like, “Let’s try to accomplish this in two or three months”, explaining why we are doing it. Then we all work on that every day.

So, a typical day involves coming to the lab, working towards clearly defined objectives for the next few months, still having an open mind if better ideas come along. Bell Labs is highly cooperative environment and we often walk into neighboring lab for fruitful discussions, mutual assistance, and a sharing of expertise, which is one of the lab’s great strengths.

**Q10: How do people decide on research topics at Nokia Bell Labs? Is it driven by personal interest, management demand, or both?**

**A10:** It’s a combination. One of Nokia Bell Labs’ strengths is its bottom-up approach, which highly values the input of researchers. Most of the time, research direction is driven by the researchers themselves, often influenced by interactions with the business but with some guidance from management.

However, there are cases where management directs the research based on business needs. These projects are more focused and are directly tied to the objectives of Nokia’s business units and almost always closely related to the researcher’s expertise. I also conduct weekly meetings with the business division to understand their priorities and needs and provide expertise.

Publication is important for us, too, but meeting the immediate needs of the business and securing patents can take precedence. Some projects are developed solely for internal use and may not be published externally for a long time, if at all. This is one of the key differences from academia, where the emphasis is usually on publishing results.

**Q11: How has Nokia Bell Labs evolved, and what changes have you witnessed?**

**A11:** That’s a question we often receive, and the answer really depends on the specific area. For instance, in material science, Bell Labs used to have a significant presence, but this has been reduced over time. People from this field might view Nokia Bell Labs as having downsized, but from the perspective of Nokia’s core business—telecommunication equipment, fiber optics transmission systems, wireless communication systems, and data networking—Nokia Bell Labs’ research has become more focused. For example, our work in optical systems is as robust now as it was 25 years ago. However, certain areas like fiber fabrication, where we once had an entire division, have been divested.

Nokia Bell Labs has evolved by concentrating more on the active business areas, but it also continues to invest in new areas that might not have immediate commercial applications. For example, we are actively involved in research on quantum computing, including topological quantum computing, despite not having a direct business interest in it yet. Our researchers have even received the Nobel Prize for their research on the fractional quantum Hall effect, closely related to our quantum computing effort.

**Q12: You joined Bell Lab at a golden time in 1997, surrounded by many brilliant people. For example, Prof. John Bowers and Prof. Evelyn Hu have also been the light people in our previous interviews. During your time at Bell Labs so far, did anyone leave a particularly strong impression on you?**

**A12:** Indeed, I met many inspiring people at Bell Labs, which I consider a great privilege. James Gordon, for instance, was someone I initially knew only through his work on nonlinearity in fibers. Learning more about his pioneering efforts on incorporating quantum effects in optical communication and the Gordon-Holevo capacity limit revealed the depth of his intellect, which was extraordinary. On a personal level, James was also fantastic. He has the best sense of humor of anybody I know. After his passing, I learned from his wife that he rarely used books, preferring to derive everything from first principles, a rare skill today.

Andy Chraplyvy, who hired me, also left a lasting impression. As my first boss, he brought me to the business unit early on, helping me understand the commercial aspects vital to our work. His guidance was crucial in shaping my approach to research and scientific writing.

Then there is Gerard Foschini, known for inventing MIMO, who also made contributions to optics, particularly in polarization-mode dispersion. Working with him not only expanded my knowledge in fiber capacity but also helped me approach problems effectively, it’s also what I learned from all these people.

I was also so fortunate to interact with Arthur Ashkin, another mentor. He had an incredible imagination. For instance, he invented optical tweezers, which earned him a Nobel Prize in 2018. I learned from him that many areas we assume are fully understood still hold undiscovered secrets. His ability to rethink “settled matters” and uncover new insights was truly inspiring. For instance, with the invention of the optical tweezers, he saw the potential in focusing a laser in a way that no one else had considered. This ability to look deeply into problems and find new approaches was one of his greatest strengths.

**Figure:**
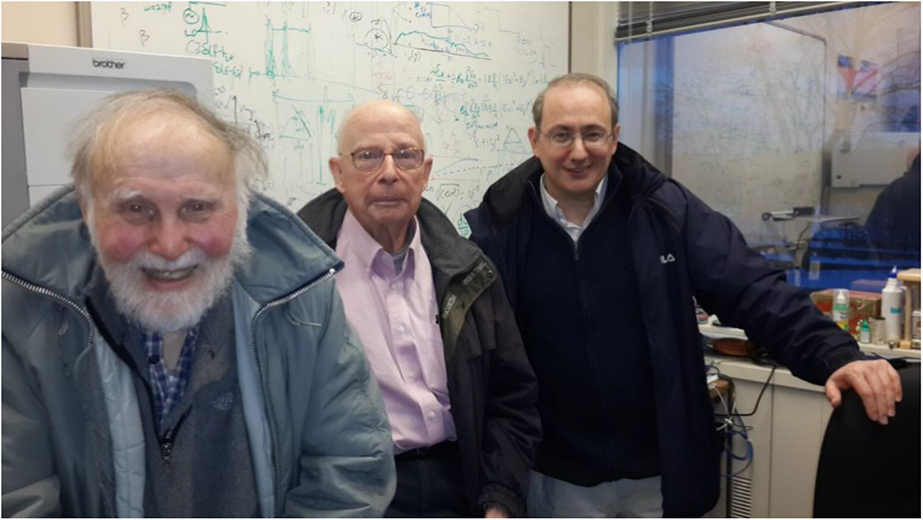
Group photo of Arthur Ashkin (left), Gary Boyd (middle) and René-Jean Essiambre (right) in René’s office at the Crawford Hill Laboratory in Holmdel, New Jersey

**Q13: You presented the Nobel lecture on Arthur Ashkin’s behalf in 2018. Why did he choose you to present this Nobel physics prize lecture for him, and could you share more insights from this experience?**

**A13:** Sure, it was a great honor that he asked me to give the Nobel lecture on his behalf. I got to know him around 2000, but we were not initially close. Our friendship deepened significantly in the early 2010s through many detailed discussions. We lived nearby, about a six-minute drive apart, and he often invited me over. We spent countless hours in his downstairs lab, talking and discussing various topics, and he gave me his book on optical tweezers to read. It’s truly remarkable to become close friends with such a great scientist.

When Art, Arthur's nickname used by his colleagues, won the Nobel Prize, he initially asked if I could help him with the presentation, from formatting PowerPoint slides to ensuring it reflected his intentions accurately and providing feedback. Later, due to health reasons, he couldn’t travel to Stockholm to deliver the lecture himself. So, he asked me if I could give the lecture on his behalf in Stockholm, which I found both an honor and quite stressful—I wanted to give the best possible presentation for him. I dedicated two months to preparing, covering everything from the Nobel Lecture content to logistics for the trip.

Throughout this process, I learned a great deal from him. Even though my direct research wasn’t on optical tweezers, the years spent discussing them with him equipped me well to assemble the presentation. There were many memorable moments. For instance, the original videos for the lectures were on 30-year-old VHS tapes, stored near strong magnets and never looked at. Despite concerns about their quality, we digitized them and they turned out wonderfully for the presentation. It was a once-in-a-lifetime experience, incredibly fulfilling both personally and professionally.

**Q14: You have done many great works in optical communications. What advice would you offer to young scientists or engineers just starting in their career in this field?**

**A14:** Giving advice carries a big responsibility because I want to guide young professionals without steering them in the wrong direction. What I can say is that the topic you’re going into should have potential for future growth, even though it’s difficult to predict the future of any specific topic and the unexpected can and sometimes does happen. The people you study with and work for—your advisors, mentors and so on—are crucial. Inspiring mentors can make a profound difference in your career development. It’s important to surround yourself with people from whom you can learn and who motivate you although it may not be easy to identify these individuals at first.

When starting graduate school or launching a career, you often don’t know exactly whom you’ll be working with or what opportunities will arise. Many young students and researchers, including myself, would doubt that there’s still anything left to discover. I’ve experienced this doubt several times in my career, thinking that certain fields had reached their limits. But it’s rarely true. There’s almost always something more to uncover and new revolutions in the field can occur. In optical communication, for instance, we’re close to the nonlinear Shannon limit in commercial systems, which might suggest we’ve reached the capacity limits of fiber. However, multicore and novel hollow-core fibers came along. It’s important not to give up hope and continue to pursue innovative ideas. At the same time, if a particular approach yields no results over time, it’s essential to be flexible and willing to explore new directions, while always keeping an open mind. There are countless discoveries waiting to be made and so much more to explore.

**Q15: As a past president of the IEEE** **Photonic Society, could you share some insights from your experience in service and outreach? How do we balance the huge requirement for our research with contributing to society as much as possible?**

**A15:** Volunteering for a society can be very rewarding. But as a young researcher, you may already juggle many responsibilities while trying to establish your career. The decision to volunteer can vary greatly among individuals—some may choose to start early, while others might prefer to wait until they’re more established. My suggestion is to follow what you feel. If you feel compelled to volunteer as a young researcher, you should go for it, but always be mindful of managing your time well, balancing your commitments effectively to ensure you continue to produce significant work and advance in your career. Engaging in volunteer work can be very enlightening, especially for young researchers. Your connection with graduate students can also encourage them to get involved. Volunteering is a good opportunity to figure out how professional societies function, which may appear somewhat mysterious for a graduate student.

I served as the president of the IEEE Photonics Society for two years. Although it took up a lot of my time, I’m glad I did it. On the other hand, I also enjoy research, so it’s time for me to focus more on that. My experience in the Photonics Society has given me a better understanding of how it works and has helped me assist others. I now have a better sense of what’s important to people in different sectors—industry, academia, and young professionals. I believe this experience makes me a better ambassador for photonics in general.

**Figure:**
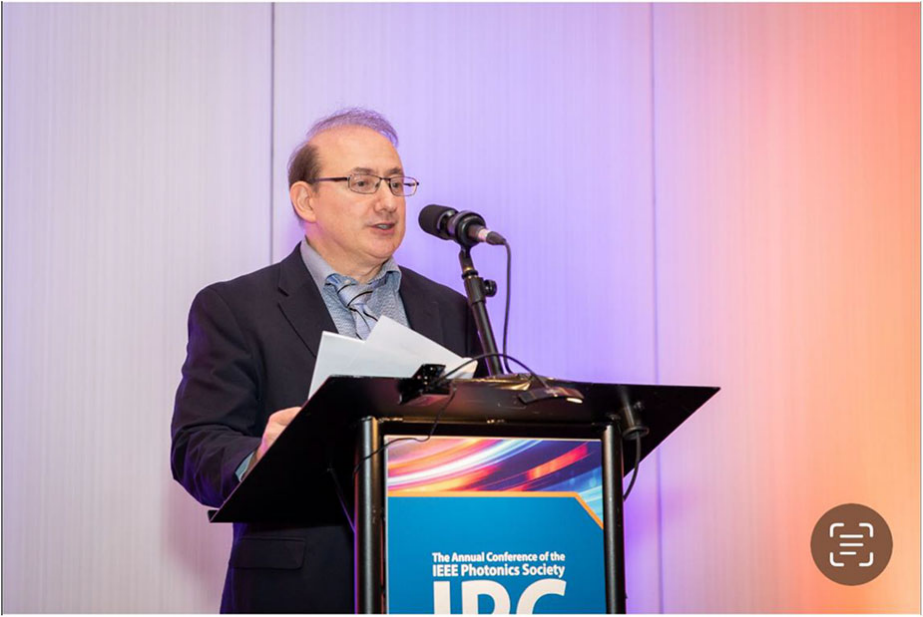
Dr. René-Jean Essiambre hosting an IEEE Photonics Society event

**Q16: Could you share more about your strategies to promote photonics during your presidency**.

**A16:** Certainly. While photonics has been successful, it often faces challenges in gaining wider recognition. As the president, I focused on enhancing the understanding of photonics among the general public, specialists, and government officials. For instance, the backbone of the internet heavily relies on optical fiber, which is a key element of photonics, along with other optical components like lasers. I believe that sometimes the field hasn’t received enough exposure from professional societies. My approach was to simplify our explanations of what photonics is and to highlight its contributions to society—both historically and in emerging areas like integrated optics and optical quantum technologies. Ensuring widespread awareness about the current and potential impact of photonics was a key priority during my presidency.

**Q17: Growing up in a remote fishing village, how did your upbringing and family life shape your career and personal philosophy?**

**A17:** I grew up in a small village in Canada where fishing and lumberjacking were the main industries and almost everyone spoke French only. I was raised in a large family, with seven children in my immediate family and a wealth of cousins—78 first cousins, to be exact. This environment taught me how to interact with people from a young age.

As the youngest boy in my family, I was raised primarily by my four sisters while my parents ran a grocery store. I learned a lot from my sisters, in particular the strength of collaborative work. Despite having no close family members in the field of science—except for one uncle whom I met only once —I developed a keen interest in science during my childhood. My family was very supportive, encouraging me to pursue my interests freely.

Coming from a remote and cold region in northeast Canada taught me the importance of community values. In such an environment, you learn the necessity of relying on others for survival. The sense of community was exceptionally strong—we knew and helped each other, including extended relatives and community members in difficult situations. This has deeply influenced my personal philosophy and career, emphasizing the value of community, collaboration and fairness.

**Q18: Aside from your professional interest, how do you relax? What hobbies or activities do you enjoy?**

**A18:** I was raised in the wilderness in some way, so I’ve always enjoyed outdoor sports like cycling and running, as well as soccer and tennis. I also enjoy training in the gym. These sports are my favorites. As for hobbies, I have a keen interest in history. I believe there’s much to learn from history, although it may not always be entirely accurate. For instance, in the history of science, some discoveries go unnoticed or are later credited to others. So, I approach history with a critical eye, but I find it fascinating, nonetheless. I also have a mild interest in geography.

**Q19: With the proliferation of scientific journals in recent decades, what are your views as a scientist on what constitutes a good journal, or even a top journal?**

**A19:** That’s a great question. With the volume of articles being published today, navigating through them is increasingly challenging. Our reading speed hasn’t increased much in the past 50 years, yet publications have grown dramatically. So, how do we manage this? In my view, a good journal is defined by the quality and significance of its research. The merit of science should take precedence, regardless of whether the topic is currently popular or widely recognized. There’s a tendency to prioritize ‘hot topics’ due to funding or popularity, but this doesn’t always reflect the true importance. A publication should be judged on its contribution to advancing the field in a rigorous and meaningful way.

Scientific quality stands the test of time. Sometimes, an article’s true significance is recognized long after its initial publication. This enduring quality is what distinguishes a noteworthy publication. Personally, I often revisit early quantum research papers, some nearly a century ago. Their enduring insights offer substantial value and often remain relevant today, illustrating the lasting impact of high-quality research.

## Supplementary information

Supplementary Video-Full Interview

